# The effects of 8-week wearable resistance training on neuromuscular adaptations in soccer players

**DOI:** 10.3389/fphys.2026.1828654

**Published:** 2026-07-28

**Authors:** Bo Tang, Mingyue Hou, Rui Yang

**Affiliations:** 1 Department of Physical Education, Hohai University, Nanjing, China; 2 Business School, Hohai University, Nanjing, China

**Keywords:** neuromuscular adaptations, power, rate of force development, soccer performance, wearable resistance

## Abstract

**Purpose:**

Although wearable resistance training (WRT) has been shown to enhance athletic performance, the underlying neuromuscular and morphological mechanisms remain poorly understood. This study investigated the effects of an 8-week in-season WRT program on physical performance and neuromuscular adaptations in collegiate soccer players.

**Methods:**

Thirty highly trained male soccer players were stratified and randomly assigned to a WRT group (n = 15; age: 22.8 ± 2.9 years; height: 180.1 ± 6.2 cm; body mass: 76.1 ± 7.4 kg; 2% body mass load distributed on the lower limbs) or a control group (CON, n = 15; age: 22.2 ± 3.3 years; height: 178.7 ± 5.5 cm; body mass: 74.3 ± 6.5 kg; unloaded placebo garments). Participants completed three weekly intervention sessions over an 8-week period, integrated into their regular technical-tactical training. Pre- and post-intervention assessments included 10-m and 30-m sprint times, countermovement jump (CMJ) height, 5–0–5 change-of-direction (COD) time, maximal voluntary contraction (MVC) torque, knee extensor isometric rate of force development (RFD), normalized vastus lateralis RMS amplitude, and ultrasound-derived vastus lateralis architecture (muscle thickness and pennation angle).

**Results:**

Mixed-design ANOVA revealed that the WRT group demonstrated significantly greater improvements than CON in 10-m (Cohen’s d = -1.18, p = 0.025) and 30-m sprint times (d = -0.85, p = 0.016), CMJ height (+10.97%, p < 0.001), and 5–0–5 COD performance (d = -0.95, p = 0.027). Importantly, WRT elicited a significant increase in RFD (+7.95%, d = 1.08, p = 0.049). However, no significant group × time interactions were observed for MVC, sEMG amplitude, or muscle morphology (all p > 0.05). Session-RPE training loads did not differ between groups.

**Conclusion:**

Low-load WRT (2% body mass) is an effective modality for enhancing multidirectional explosive performance. The increase in RFD without concomitant changes in maximal strength or muscle morphology suggests that these functional gains are primarily driven by early-phase neural adaptations rather than structural hypertrophy. Practically, WRT represents a time-efficient in-season micro-dosing strategy that may augment explosiveness without significantly increasing perceived internal training load.

## 1 Introduction

Soccer is a physically demanding, multidirectional sport characterized by intermittent high-intensity actions, including maximal sprinting, jumping, and rapid changes of direction, interspersed with periods of lower-intensity recovery (Hammami et al., 2018). A recent time-motion analysis of English Premier League goal-scoring sequences showed that the most common movements preceding goals were linear advancing actions (32.4 ± 1.0%), decelerations (20.2 ± 0.9%), and turns (19.8 ± 0.9%), with at least one high-intensity movement observed in 82.9 ± 1.5% of player involvements (Martínez-Hernández et al., 2023). Success in these decisive game actions relies heavily on well-developed neuromuscular capacities, particularly explosive strength and the ability to generate force rapidly (Morgans et al., 2018). Consequently, strength and conditioning professionals are continually seeking effective and time-efficient training modalities to augment these physical qualities, especially during the congested in-season period, when technical-tactical preparation takes precedence and mitigation of residual fatigue is critical (Loturco et al., 2022).

Traditional resistance training (RT), commonly performed through structured gym-based exercises using free weights, machines, or other external loads, remains a fundamental method for developing the neuromuscular system and enhancing athletic performance (Rong et al., 2025). Its training stimulus is typically manipulated through variables such as external load, training volume, exercise selection, contraction mode, movement velocity, and inter-set recovery, allowing practitioners to target maximal strength, explosive strength, power, and force-production capacity. This view is supported by a recent systematic review and meta-analysis comparing weightlifting training, traditional RT, and plyometric training, which indicated that resistance-based training methods can improve strength-, power-, and speed-related outcomes in athletic population (Morris et al., 2022). The strength and power adaptations elicited by RT are underpinned by a combination of neural and morphological factors (Moritani, 1993). Early-phase neural adaptations predominantly involve enhanced motor unit recruitment, increased firing frequencies (rate coding), improved intermuscular coordination, and reduced antagonist co-activation (Ozmun et al., 1994; Häkkinen et al., 2000). Conversely, morphological adaptations, such as increases in muscle size and changes in ultrasound-derived architectural measures, typically require prolonged periods of consistent, high-mechanical-tension training (McCarthy et al., 2002; Häkkinen et al., 2003). These adaptations provide the physiological basis for using traditional RT to improve strength- and power-related qualities relevant to sprinting, jumping, acceleration, deceleration, and change-of-direction performance.

Although gym-based RT is effective, there is a growing shift towards modalities that provide a more ecologically valid and sport-specific stimulus. Wearable resistance training (WRT), which involves attaching small external loads directly to the limbs, trunk, or other body segments during dynamic movement tasks has emerged as a promising intervention (Li S. et al., 2026). Unlike traditional gym-based RT, WRT allows athletes to perform sprinting, jumping, acceleration, deceleration, and change-of-direction tasks under light external loading conditions while maintaining movement patterns that closely resemble sport-specific actions. The primary biomechanical rationale for WRT is its capacity to provide a movement- and vector-specific overload during unconstrained, sport-specific movements, thereby increasing the mechanical and neuromuscular demands imposed on the active musculature during task-specific locomotion and facilitating efficient transfer of training adaptations to athletic performance (Bustos et al., 2020). By using relatively light external loads, commonly ranging from approximately 1% to 5% of body mass, current acute evidence suggests that WRT may provide a movement-specific overload while minimizing immediate disruption to movement mechanics (Bartosz-Jeffries et al., 2025). However, whether repeated or long-term WRT exposure preserves or alters sport-specific movement patterns remains unclear and warrants further investigation.

Consistent with this rationale, previous studies have shown that WRT can acuyely metabolic, kinematic, and kinetic responses during walking, running, sprint running, and jumping tasks, suggesting its potential as a movement-specific overload strategy (Macadam et al., 2017). In soccer players, lower-limb WRT loads of 1%–5% of body mass have been shown to acutely influence change-of-direction performance, with load placement affecting the magnitude of the response (Istvan Rydså and van den Tillaar, 2020). Moreover, during small-sided soccer games, very light lower-limb wearable resistance micro-loads have been reported to attenuate the decline in repeated change-of-direction performance (Ltifi et al., 2023). From an applied soccer perspective, these characteristics make WRT particularly relevant because it can be integrated into field-based drills that include accelerations, decelerations, sprinting, jumping, and rapid changes of direction, thereby providing an additional resistance stimulus without substantially altering the ecological context of training. However, despite evidence supporting its performance benefits, the neuromuscular mechanisms underpinning these improvements remain largely unclear, as previous WRT research has predominantly focused on functional performance, metabolic, kinematic, and kinetic outcomes rather than direct neuromuscular or morphological adaptations (Macadam et al., 2017; Brown et al., 2022; Bertochi et al., 2024). Most previous research has focused on functional outcomes, leaving limited information regarding maximal force production, rate of force development (RFD), neural drive assessed via electromyography, and muscle architectural characteristics following WRT interventions (Istvan Rydså and van den Tillaar, 2020; Brown et al., 2022; Ltifi et al., 2023; Brown et al., 2024).

Clarifying these mechanisms underpinnings is essential for optimizing exercise prescription. If low-load WRT primarily induces early-phase neural adaptations rather than structural hypertrophy, it may represent a practical in-season stimulus by providing a neural-oriented overload within regular field-based training. However, whether such an approach reduces muscle damage, central fatigue, or recovery demands compared with heavier resistance training requires direct investigation using appropriate physiological and recovery markers (Siddique et al., 2020; Rong et al., 2025). Moreover, identifying stimuli capable of eliciting further adaptation in highly trained athletes, who often approach their physiological ceiling, is of considerable practical importance (Aslam et al., 2025). Therefore, a comprehensive multi-level assessment encompassing both performance outcomes and physiological adaptations is necessary to validate the application of WRT in trained athletes.

This study aimed to address this gap by investigating the effects of an 8-week in-season WRT program on a comprehensive set of neuromuscular and physical performance variables in collegiate soccer players. We hypothesized that, compared with a control group performing identical training with unloaded placebo garments, the WRT group would demonstrate greater improvements in sprint performance, jump height, and agility. We further hypothesized that these functional gains would be primarily driven by neural adaptations, specifically increased RFD, while showing minimal changes in maximal isometric strength (MVC) and muscle architecture, consistent with the high-velocity and low-load characteristics of the intervention.

## 2 Methods

### 2.1 Experimental approach to the problem

This study employed a stratified, randomized, single-blind, controlled trial design to investigate the effects of an 8-week in-season WRT program on neuromuscular adaptations, muscle morphology, and physical performance in collegiate soccer players. Following baseline assessments, participants were stratified and randomly allocated to either the WRT group or an unloaded control (CON) group. Assessments were conducted 48 h prior to the intervention (Pre) and 48 h after the final training session (Post). To minimize circadian influences, all testing sessions were standardized to the same time of day (16:00–18:00 h). Participants were instructed to abstain from vigorous physical activity and alcohol consumption for 24 h prior to testing and to maintain their habitual dietary intake. The study protocol was approved by the Institutional Review Board at Hohai University (approved number: HHU-TYX-202601), and written informed consent was obtained from all subjects in accordance with the Declaration of Helsinki.

### 2.2 Subjects

Sample size was determined *a priori* using G*Power (version 3.1; Heinrich Heine University Düsseldorf, Germany). The calculation was conducted using the F-test family with the statistical test specified as “ANOVA: repeated measures, within–between interaction,” corresponding to the primary group × time interaction in the present 2-group × 2-time mixed-design ANOVA. The following parameters were used: a medium effect size of f = 0.25, an alpha level of 0.05, a statistical power of 0.80, two groups, two repeated measurements. This calculation indicated that a minimum total sample size of 28 participants was required. To account for potential attrition, 30 male collegiate soccer players were recruited. All participants were active varsity team members and were classified as Tier 3 (highly trained or national level) athletes according to the participant classification framework of McKay et al. (2022) (McKay et al., 2022). To ensure baseline comparability, participants were stratified by playing position (defender, midfielder, forward) and baseline 30m sprint time, before random allocation. Baseline 30-m sprint time was selected because it was considered a representative field-based indicator of overall linear sprint ability and was consistent with routine team testing practice. Given the relatively small sample size, stratification based on multiple outcome variables was avoided to reduce the risk of over-stratification. Participants were then randomly allocated to either the WRT group (WRT, n = 15; age: 22.8 ± 2.9 years; height: 180.1 ± 6.2 cm; body mass: 76.1 ± 7.4 kg; soccer playing experience: 8.3 ± 2.3 years) or the control group (CON, n = 15; age: 22.2 ± 3.3 years; height: 178.7 ± 5.5 cm; body mass: 74.3 ± 6.5 kg; soccer playing experience: 7.8 ± 2.5 years). Inclusion criteria were: (1) at least 5 years of competitive soccer training experience; (2) a current training frequency of at least five sessions per week; (3) absence of lower-limb musculoskeletal injury within the preceding 6 months; and (4) no use of performance-enhancing substances. Goalkeepers and players performing concurrent structured resistance training were excluded.

### 2.3 Training intervention

The 8-week intervention was conducted during the competitive season. Both groups followed an identical team training regimen consisting of four on-field sessions and one official match per week. The experimental intervention was administered three times weekly (Monday, Wednesday, Friday) and was embedded within regular technical-tactical sessions to minimize disruption.

During the intervention, the WRT group wore commercially available compression shorts (Sportboleh Sdh Bhd, Malaysia) equipped with anterior and posterior thigh pockets, together with weighted calf sleeves. The external load corresponded to 2% of each individual’s body mass (BM), symmetrically distributed across the lower limbs, with 1% BM applied to the proximal thighs and 1% BM applied to the distal calves. To integrate the neuromuscular stimulus within the regular training schedule while managing overall training demands, WRT was performed in a ~30-min block immediately after a standardized 15-min warm-up and prior to the main training session. The warm-up consisted of light jogging, dynamic stretching, and lower-limb activation exercises. The intervention was soccer-specific and primarily included acceleration drills, such as repeated short-distance starts and progressive accelerations; deceleration drills, such as sprint-to-stop actions, braking tasks, and stop-and-go movements; change-of-direction exercises, such as shuttle runs, zig-zag runs, cutting maneuvers, and 180° turning actions; and small-sided games involving repeated acceleration, deceleration, re-acceleration, and multidirectional movements, which are commonly used in soccer-specific training to improve physical conditioning, technical actions, and match-related movement demands (Katis and Kellis, 2009). The selected loading magnitude was based on previous work indicating that loading of approximately 2% body mass can provide an effective overload stimulus without meaningfully altering movement mechanics (Feser et al., 2020). The detailed weekly training prescription is provided in **Supplementary Table 1**.

The CON group performed identical training content for the same duration and at the same time points as the WRT group but wore placebo garments matched in appearance and material and containing no added mass. This approach aimed to blind participants to group allocation as far as possible and to control for psychological expectancy and proprioceptive effects associated with wearing additional equipment.

To ensure that the primary between-group difference was attributable to the wearable resistance stimulus, internal training load was monitored for all sessions using the session rating of perceived exertion (sRPE) method (Impellizzeri et al., 2004). Participants reported a single RPE score (0–10 scale) 30 min after each session, and training load was calculated as sRPE (arbitrary units, AU) = RPE × session duration (min).

### 2.4 Testing procedures

Testing was conducted over two sessions, both pre- and post-intervention, separated by 48 h. Session one consisted of performance testing, and session two comprised laboratory-based neuromuscular assessments. Physical performance tests were conducted in a fixed order for all participants at both Pre and Post assessments: countermovement jump, 10-m and 30-m linear sprint testing, and 5–0–5 change-of-direction testing. This order was selected to minimize fatigue-related interference, and the same order, warm-up procedures, verbal instructions, and rest intervals were used at Pre and Post. These assessments were administered by trained assessors with an academic background in sports science and practical experience in field-based performance testing and laboratory-based neuromuscular assessment. Before data collection, the assessors were familiarized with the standardized procedures, including participant instructions, warm-up procedures, trial execution, rest intervals, and data recording. The same assessors conducted both the pre- and post-intervention assessments to minimize inter-tester variability. All assessments were performed at the same time of day to minimize diurnal variation. The intra-class correlation coefficients (ICC) for all performance tests ranged from 0.88 to 0.94.

#### 2.4.1 Physical performance tests

##### 2.4.1.1 Linear sprint performance

Linear sprint speed was assessed over 10m and 30m on a standardized indoor court using a dual-beam photoelectric timing gate system (Witty, Microgate, Bolzano, Italy). Timing gates were positioned at 0 m, 10 m, and 30 m, and tripod height was standardized to approximately hip level to prevent premature triggering by upper-limb motion or trunk lean. Participants adopted a stationary split-stance position with their preferred leg forward, with the toe of the front foot positioned 0.5 m behind the first timing gate. A volitional start was used to eliminate reaction time, and participants sprinted maximally beyond the final 30 m gate without premature deceleration. Each participant completed three maximal trials with 3 min of passive recovery. The fastest 0–10 m and 0–30 m times were retained for analysis (Haugen and Buchheit, 2016).

##### 2.4.1.2 Change-of-direction speed performance

Change-of-direction speed was evaluated using the traditional 5–0–5 test. Dual-beam timing gates were set at approximately hip height (0.9 m) to prevent false triggering from arm movement. The gates were positioned exactly 5 m from a clearly marked turning line. Participants sprinted maximally from the start line for 10 m, performed a 180° turn at the turning line, and sprinted 5 m back through the timing gates. Time was recorded between the initial and return passage through the gates. Each participant performed three trials separated by 3 min of passive recovery, and the best time was used for analysis (Sheppard and Young, 2006).

##### 2.4.1.3 Countermovement jump

Vertical jump performance was assessed using a countermovement jump on a three-dimensional force platform (Kistler, Winterthur, Switzerland) sampled at 1000 Hz. Participants stood in the center of the platform with hands on hips to eliminate arm swing. They performed a rapid eccentric countermovement to a self-selected depth followed immediately by a maximal concentric jump and maintained full lower-limb extension during flight. Jump height was calculated using the flight-time method according to the equation h = gt²/8, where g represents gravitational acceleration and t represents flight time. Three maximal CMJs were performed with 3 min of passive recovery, and the highest value was used for analysis. Intraclass correlation coefficients for performance tests ranged from 0.88 to 0.94 (Springham et al., 2024).

#### 2.4.2 Neuromuscular and morphological assessments

##### 2.4.2.1 Maximal voluntary contraction and rate of force development

Maximal voluntary isometric contraction (MVC) of the right knee extensors was measured using an isokinetic dynamometer (Humac Norm, CSMi, Stoughton, MA, USA). Participants were seated with the hip and knee flexed to 90° and instructed to push as hard and as fast as possible for 3–5 s. Rate of force development (RFD) was calculated as the steepest slope of the torque-time curve over a 50 ms rolling window. Contraction onset was defined as the point at which torque exceeded two standard deviations above baseline noise (Maffiuletti et al., 2016).

##### 2.4.2.2 Surface electromyography

Surface electromyography (sEMG) of the vastus lateralis was recorded concurrently during MVC trials using a wireless system (Trigno, Delsys, Natick, MA, USA; sampling rate 2000 Hz). The vastus lateralis was selected because it is a major contributor to knee extension torque and allowed synchronized assessment of local quadriceps activation during the standardized knee extensor isometric task. Before electrode placement, the skin was shaved if necessary, lightly abraded, and cleaned with alcohol. The sensor was positioned according to SENIAM recommendations, approximately two-thirds of the distance from the anterior superior iliac spine to the lateral border of the patella and aligned parallel to the presumed muscle fiber direction. Signal quality was checked during rest and submaximal contractions before testing.

Raw sEMG signals were band-pass filtered at 20–500 Hz using a fourth-order Butterworth filter. RMS amplitude was calculated over a 500-ms epoch centered on peak knee extension torque, using the MVC trial with the highest peak torque for analysis. RMS values were normalized to the maximal M-wave response (Mmax) elicited by percutaneous femoral nerve stimulation. Single 1-ms rectangular pulses were delivered with progressively increasing intensity until the peak-to-peak M-wave amplitude reached a plateau. Supramaximal intensity was then set above the intensity required to elicit the maximal response, and the largest peak-to-peak M-wave amplitude from three supramaximal stimuli was defined as Mmax. Vastus lateralis RMS amplitude was expressed as %Mmax (Hermens et al., 2000).

##### 2.4.2.3 Ultrasound-derived muscle thickness and pennation angle

Ultrasound-derived vastus lateralis muscle thickness and pennation angle were assessed using B-mode ultrasonography (Logiq S8, GE Healthcare, Chicago, IL, USA) with a linear-array probe, following previously reported procedures for ultrasound-based muscle morphology assessment in athletic populations (Li M. et al., 2026). Participants rested in a supine position with the knee fully extended before image acquisition. The measurement site was identified at 50% of the distance between the greater trochanter and the lateral epicondyle of the femur and was marked with a skin-safe marker to ensure consistent probe placement between Pre and Post assessments. A generous amount of ultrasound gel was applied, and care was taken to minimize probe pressure and avoid compression of the underlying muscle tissue.

The probe was positioned longitudinally along the presumed fascicle direction of the vastus lateralis. Three images were collected from the right vastus lateralis at each time point. Muscle thickness was defined as the perpendicular distance between the superficial and deep aponeuroses. Pennation angle was defined as the angle between the visible muscle fascicle and the deep aponeurosis. The mean value of the three images was used for statistical analysis. All images were acquired and analyzed by the same trained assessor using identical procedures at Pre and Post (Kwah et al., 2013).

### 2.5 Statistical analysis

Data are presented as mean ± standard deviation (SD). Normality and homogeneity of variance were assessed using the Shapiro–Wilk and Levene tests, respectively (Wang et al., 2019). Independent-samples t-tests compared baseline characteristics and weekly mean sRPE training loads between groups. A mixed-design analysis of variance (Group: WRT vs CON; Time: Pre vs Post) was used to examine intervention effects on all dependent variables. When a significant group × time interaction was detected, Bonferroni-adjusted simple-effects comparisons were performed. These follow-up comparisons examined within-group Pre-to-Post changes and between-group differences in change scores. Effect sizes for between-group differences were calculated using Cohen’s d and interpreted according to Hopkins et al.: <0.2 trivial, 0.2–0.6 small, 0.6–1.2 moderate, and >1.2 large (Hopkins et al., 2009). Statistical significance was set at α < 0.05. All analyses were conducted using SPSS 26.0 software (IBM Corp., Armonk, NY, USA).

## 3 Results

All 30 participants completed the 8-week intervention and all testing sessions, resulting in a 100% compliance rate (>95% adherence per group). Independent-samples t-tests revealed no significant baseline differences between the WRT and CON groups for any performance, neuromuscular, or morphological variable (all p > 0.05; see **Supplementary Table 1**). Furthermore, the average weekly internal training load (sRPE) did not differ significantly between the WRT (3130.5± 90.7 AU) and CON (3129.0 ± 61.0 AU) groups over the 8-week period (p = 0.961).

### 3.1 Physical performance outcomes

**Figure 1:**
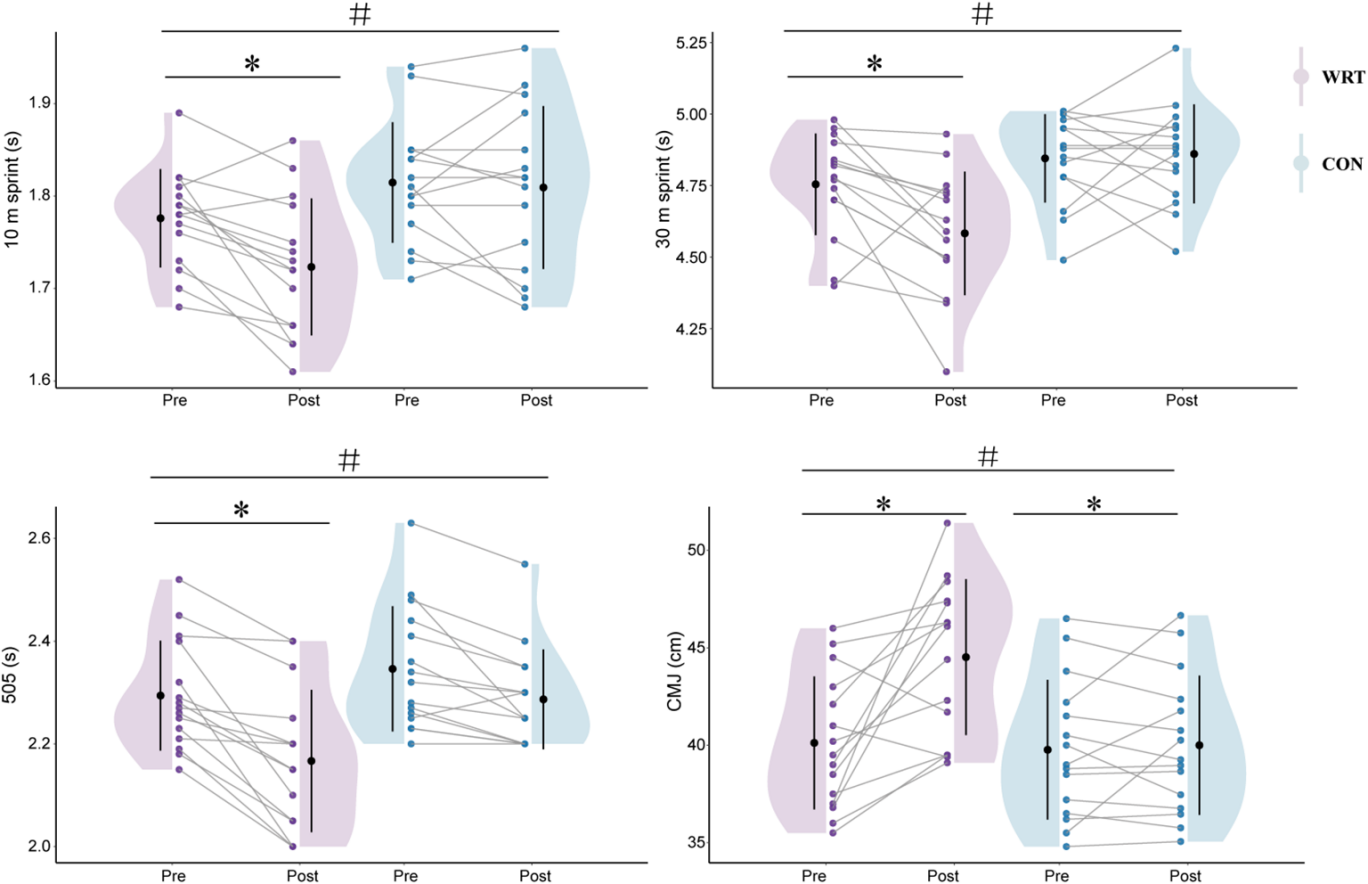
Effects of wearable resistance training on performance measures. Raincloud plots illustrate the distribution of individual values before (Pre) and after (Post) the intervention in the wearable resistance training group and the control group. Dots represent individual participants, and grey lines connect paired Pre and Post observations. The central marker and error bars indicate mean ± SD. * indicates a significant within-group Pre to Post change (p < 0.05). # indicates a significant between-group difference in the change score (Post minus Pre) (p < 0.05). **(A)** 10 m sprint time, **(B)** 30 m sprint time, **(C)** countermovement jump (CMJ) height, and **(D)** 5-0–5 change of direction (COD) time.

**Figure 2:**
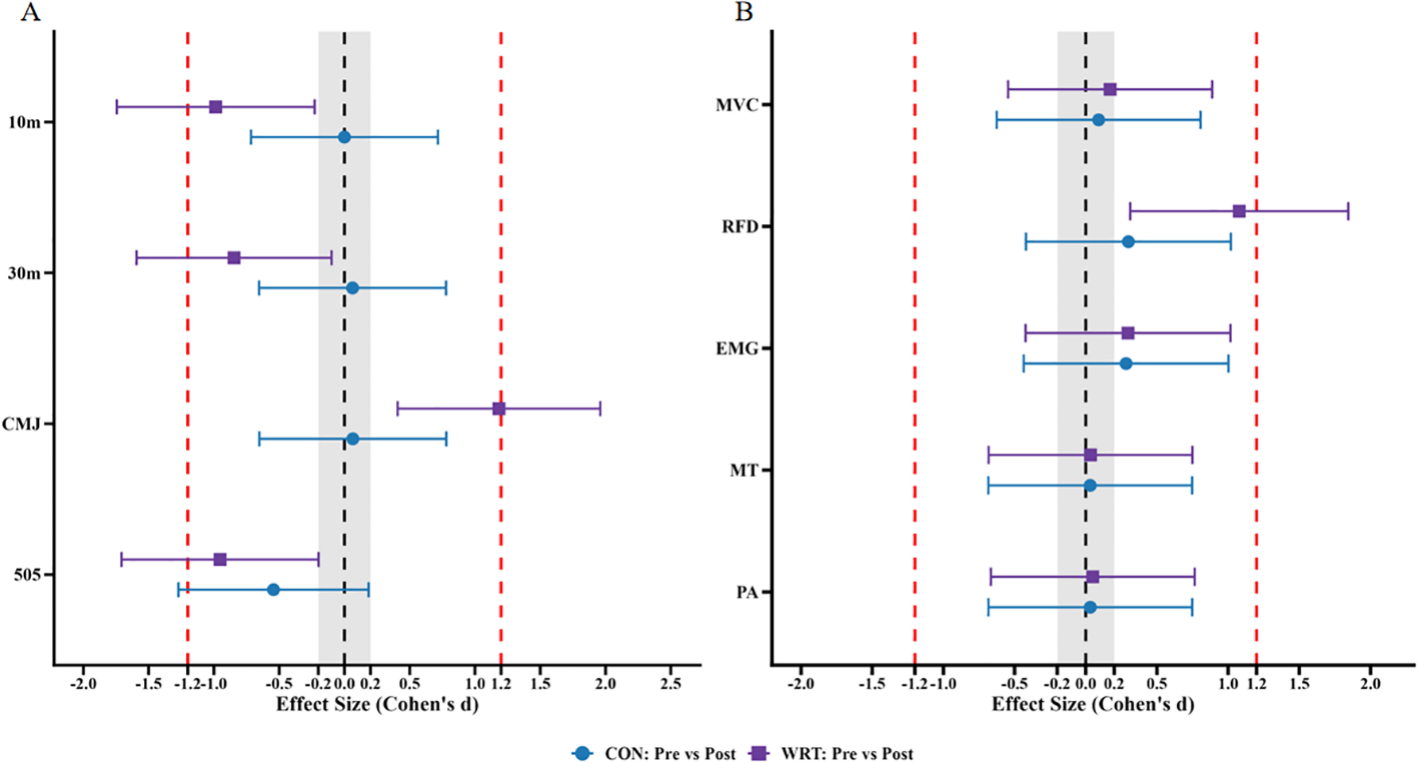
Forest plot of effect sizes on changes in performance measures and neuromuscular measures. Forest plot illustrates the standardized mean difference for the changes from before (Pre) to after (Post) the intervention. The central markers represent the effect size, and the error bars indicate the 95% confidence intervals (CIs). WRT, wearable resistance training group; CON, control group; 10m, 10 m sprint time; 30m, 30 m sprint time; CMJ, countermovement jump; 505, 5-0–5 change of direction time; MVC, maximal voluntary contraction; RFD, rate of force development; EMG, electromyography; MT, muscle thickness; PA, pennation angle. **(A)** performance measures, **(B)** neuromuscular measures.

A mixed-design ANOVA revealed significant group × time interactions across all performance metrics (**Table 1**; **Figure 1**). For the 30m sprint time, a significant interaction was observed (F (1, 28) = 6.63, p = 0.016, ηp² = 0.19). Bonferroni-adjusted simple-effects comparisons indicated a significant reduction in sprint time for the WRT group (Cohen’s d = -0.85, p < 0.01; **Figure 2**), whereas no meaningful change occurred in the CON group (d = 0.06, p = 0.770). Similarly, the 10m sprint time demonstrated a significant interaction (F (1, 28) = 5.61, p = 0.025, ηp² = 0.17), with the WRT group showing a greater improvement than the CON group (WRT: d = -1.18, p < 0.01; CON: d = 0.06, p = 0.790).

**Table 1:** Descriptive statistics and analysis of variance (ANOVA) results for performance and neuromuscular variables.

		Descriptive statistics				ANOVA P values (ηp^2^) (ηp^2^category)		
Variable	Group	Pre (Mean ± SD)	Post (Mean ± SD)	Δ (%)	ES	Time*Group	Time	Group
10m (s)	WRT	1.78 ± 0.05*	1.72 ± 0.07	-2.97	-0.99	0.03 (0.167) Large	<0.01 (0.231) Large	0.02 (0.194) Large
	CON	1.81 ± 0.07#	1.81 ± 0.09	0	0.00			
30m (s)	WRT	4.25 ± 0.18*	4.08 ± 0.22	-3.6	-0.85	0.02 (0.191) Large	0.04 (0.142) Large	<0.01 (0.282) Large
	CON	4.35 ± 0.15#	4.36 ± 0.17	0.32	0.06			
CMJ (cm)	WRT	40.12 ± 3.41*	44.52 ± 4.00	10.97	1.18	<0.01 (0.302) Large	<0.01 (0.348) Large	0.050 (0.130) Medium
	CON	39.77 ± 3.59#	40.00 ± 3.57	0.59	0.06			
5–0–5 COD time (s)	WRT	2.29 ± 0.11*	2.17 ± 0.14	-5.55	-0.95	0.03 (0.162) Large	<0.01 (0.594) Large	0.04 (0.140) Large
	CON	2.35 ± 0.12*#	2.29 ± 0.10	-2.53	-0.54			
MVC (N·m)	WRT	240.16 ± 14.91*	242.79 ± 15.82	1.1	0.17	0.39 (0.026) small	0.04 (0.150) Large	0.20 (0.058) Medium
	CON	234.20 ± 11.67	235.35 ± 13.68	0.49	0.09			
RFD (N·m·s-¹)	WRT	1077.00 ± 79.75*	1162.60 ± 79.06	7.95	1.08	0.049 (0.131) Medium	0.01 (0.295) Large	0.04 (0.147) Large
	CON	1059.34 ± 53.34#	1080.67 ± 85.38	2.01	0.26			
EMG (%Mmax)	WRT	60.04 ± 3.98*	61.30 ± 4.49	2.09	0.3	0.80 (0.002) small	<0.01 (0.252) Large	0.24 (0.048) small
	CON	58.45 ± 3.11	59.51 ± 4.28	1.81	0.28			
MT (cm)	WRT	2.53 ± 0.29	2.54 ± 0.31	0.24	0.03	0.70 (0.006) small	0.17 (0.066) Medium	0.90 (0.001) small
	CON	2.52 ± 0.33	2.53 ± 0.31	0.35	0.03			
PA (°)	WRT	14.95 ± 1.60	15.03 ± 1.64	0.54	0.05	0.71 (0.005) small	0.16 (0.069) Medium	0.88 (0.001) small
	CON	14.87 ± 1.57	14.92 ± 1.57	0.31	0.03			

Data are presented as mean ± standard deviation (SD); WRT, Wearable Resistance Training group; CON, Control group; ES, Effect Size. *Significant differences (p < 0.05) between pre-and post-test; ^#^Significant differences (p < 0.05) between EXP-and CON-group.

For vertical jump performance, CMJ height showed a strong group × time interaction (F (1, 28) = 14.96, p < 0.001, ηp² = 0.30). CMJ height increased by 10.97% in the WRT group compared with a negligible 0.59% change in the CON group. Change-of-direction speed performance assessed by the 5–0–5 test also revealed a significant interaction (F (1, 28) = 5.43, p = 0.027, ηp² = 0.16), indicating a greater reduction in completion time in the WRT group (d = -0.95, p < 0.01) than in the CON group (d = -0.54, p = 0.010).

### 3.2 Neuromuscular and morphological adaptations

**Figure 3:**
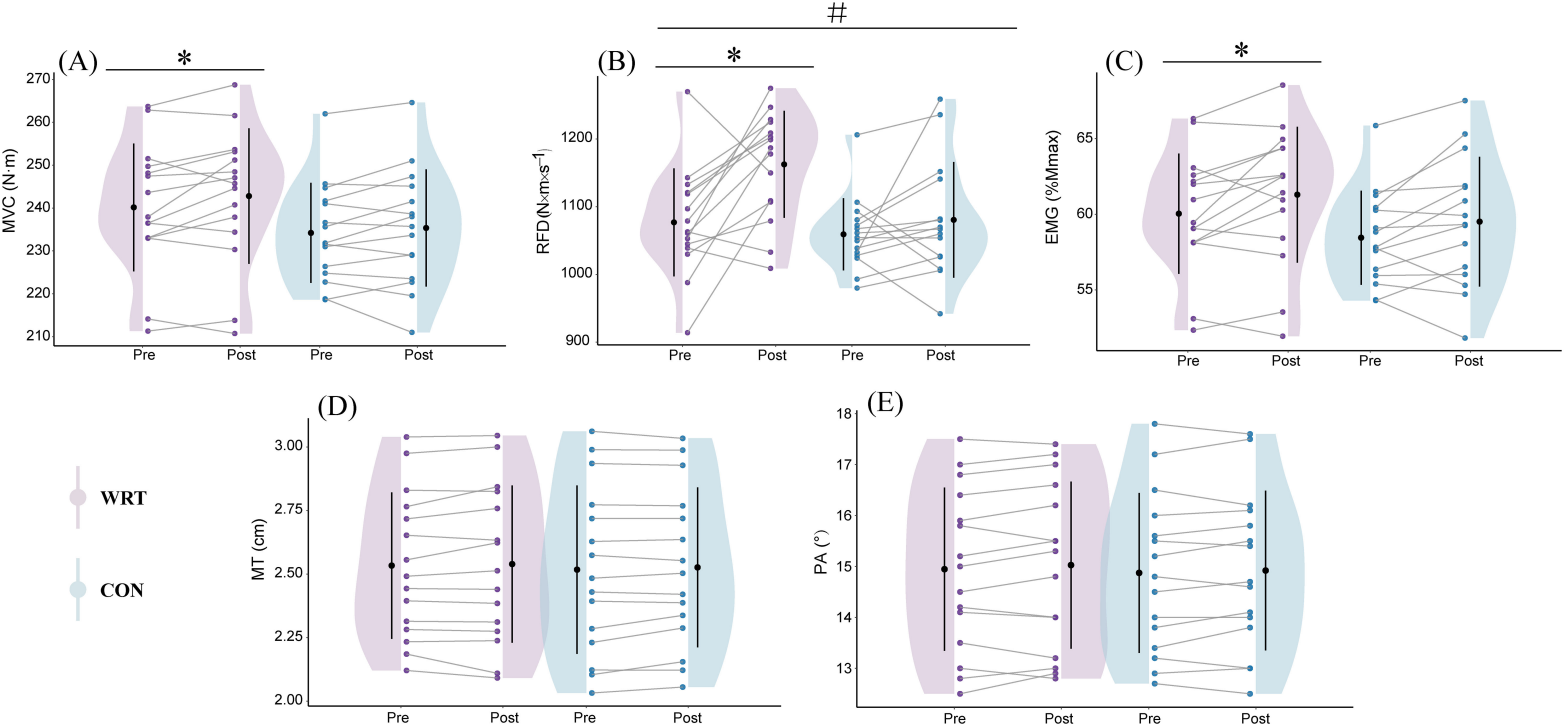
Effects of wearable resistance training on neuromuscular measures. Raincloud plots illustrate the distribution of individual values before (Pre) and after (Post) the intervention in the wearable resistance training group and the control group. Dots represent individual participants, and grey lines connect paired Pre and Post observations. The central marker and error bars indicate mean ± SD. * indicates a significant within-group Pre to Post change (p < 0.05). # indicates a significant between-group difference in the change score (Post minus Pre) (p < 0.05). **(A)** maximal voluntary contraction (MVC), **(B)** rate of force development (RFD), **(C)** electromyography amplitude, **(D)** muscle thickness (MT), and **(E)** pennation angle (PA).

For neuromuscular function, a significant group × time interaction was observed for the RFD (F (1, 28) = 4.23, p = 0.049, ηp² = 0.13; **Table 1**; **Figure 3**). Bonferroni-adjusted simple-effects comparisons confirmed a significant Pre-to-Post increase in knee extensor isometric RFD in the WRT group (+7.95%; d = 1.08, p < 0.01; **Figure 2**), with no meaningful change in the CON group (d = 0.26, p = 0.340). In contrast, maximal voluntary contraction (MVC) and normalized sEMG amplitude (vastus lateralis RMS) showed no significant group × time interactions (all p > 0.05). Although both groups demonstrated slight increases over time, no significant between-group differences were detected post-intervention. Ultrasound measurements indicated that vastus lateralis muscle thickness and pennation angle remained stable following the 8-week intervention. No significant group × time interactions or main effects of time were observed for muscle thickness or pennation angle in either group (all p > 0.05; **Table 1**).

## 4 Discussion

The primary aim of this study was to determine the effects of an 8-week in-season WRT program on physical performance and neuromuscular adaptations in collegiate soccer players. In line with our hypotheses, the WRT group demonstrated superior improvements in 10m and 30m sprint performance, CMJ height, and 5-0-5 COD times compared with the control group. Importantly, these performance enhancements were accompanied by a significant increase in RFD despite no concurrent changes in MVC, sEMG amplitude, or muscle architecture (muscle thickness and pennation angle). Collectively, these findings indicate that low-load WRT (2% body mass) is an effective stimulus for enhancing explosive performance in trained athletes and that the improvements are primarily mediated by neural adaptations rather than morphological changes.

The robust improvements across all dynamic performance metrics align with the growing body of literature supporting the efficacy of WRT in field-based sports (Wei et al., 2025; Li S. et al., 2026). Previous studies have reported that lower-body wearable resistance and soccer-specific resisted training can improve sprint-related performance in soccer players, particularly during short-distance acceleration tasks. Similarly, recent evidence suggests that resisted or soccer-specific high-intensity training may enhance COD performance by improving athletes’ ability to decelerate, reaccelerate, and produce force rapidly in multiple directions. However, improvements in vertical jump performance following WRT appear to be less consistent across studies, possibly because jump adaptations are influenced by training duration, load placement, exercise selection, and the athletes’ training background. Therefore, the present findings extend previous research by showing concurrent improvements in short-distance sprint performance, CMJ height, and 5-0–5 COD performance following an in-season lower-limb WRT intervention. Acceleration and maximal velocity are strongly governed by the ability to apply large mass-specific horizontal forces during very short ground contact times (Morin et al., 2011; Rabita et al., 2015). By applying a constant micro-load during soccer-specific ballistic movements, WRT likely increased the neuromuscular demand of repeated acceleration and sprinting actions, thereby enhancing the athletes’ ability to generate and orient force effectively during unloaded sprinting (Bustos et al., 2020). Similarly, the improved 5-0–5 COD performance may be explained by enhanced braking capacity, eccentric control, and rapid re-acceleration ability, all of which are critical determinants of change-of-direction performance (Sheppard and Young, 2006). The improvement in CMJ height may also be related to enhanced lower-limb power production and more efficient stretch-shortening cycle function, as vertical jumping performance depends on the rapid generation of force and power during dynamic multi-joint extension tasks (Cormie et al., 2011; Loturco et al., 2017).

The most compelling mechanistic finding of this study is the significant improvement in RFD in the WRT group. RFD reflects the neuromuscular system’s capacity to generate force rapidly during the early phase of contraction (< 200 ms) and is a well-established determinant of explosive athletic performance (Häkkinen et al., 2003). The present finding is in line with recent neuromuscular literature indicating that RFD is a sensitive marker of explosive strength and is strongly influenced by neural factors, including motor unit recruitment speed, discharge rate, and intermuscular coordination (Maffiuletti et al., 2016; Del Vecchio et al., 2019). Recent work on resistance training and motor unit behavior also suggests that changes in firing rate and motor unit behavior may partly explain improvements in rapid force production, although the magnitude of these adaptations can vary depending on training mode, contraction type, and testing task (Elgueta-Cancino et al., 2022). In soccer-specific contexts, recent training studies have similarly emphasized that strength- and power-oriented interventions may enhance explosive actions by improving RFD and intermuscular coordination (Oliver et al., 2024). Therefore, the improvement in RFD observed in the present study supports the notion that lower-limb WRT may preferentially target rapid force-generating capacity rather than maximal force production. The observation that RFD improved without a concurrent increase in MVC strongly suggests that the WRT stimulus targeted early-phase neural components of muscle function (Aagaard et al., 2002; Maffiuletti et al., 2016). Such adaptations likely involve enhanced rate coding, meaning increased firing frequency of high-threshold motor units, and improved intermuscular coordination rather than changes in maximal recruitment capacity (Moritani, 1993). This observation is consistent with the principle that early explosive strength adaptations are primarily neurologically mediated (Ozmun et al., 1994).

In contrast, the absence of significant changes in MVC and global sEMG amplitude requires careful interpretation. This finding is broadly consistent with recent neuromuscular literature indicating that changes in maximal force production and surface EMG amplitude after training are influenced by training load, contraction mode, task specificity, and the training status of participants.

First, the 2% body mass load, although sufficient to challenge the neuromuscular system during high-velocity movements, is considerably lower than the heavy loading ranges commonly recommended for maximizing maximal strength development in trained individuals (Aslam et al., 2025; Rong et al., 2025). Therefore, the present low-load WRT stimulus may have been sufficient to improve rapid, task-specific force production but insufficient to induce measurable changes in isometric MVC. Second, this discrepancy can be explained by the principle of testing specificity. The WRT intervention involved dynamic multi-joint ballistic movements, whereas MVC and sEMG assessments were conducted under isometric conditions. Adaptations from dynamic training often transfer poorly to isometric testing due to differences in motor unit recruitment strategies and muscle fascicle behavior between dynamic and static contractions (Schoenfeld et al., 2021). Moreover, global sEMG amplitude may lack the temporal resolution needed to detect phase-specific alterations in motor unit firing patterns associated with RFD improvements (Okudaira et al., 2023). Thus, the unchanged MVC and global sEMG amplitude do not necessarily contradict the observed improvement in RFD but rather suggest that the primary adaptation to lower-limb WRT was more likely related to rapid, task-specific neuromuscular function than to maximal isometric force capacity.

As anticipated, muscle architecture remained stable following the intervention. Muscle hypertrophy is a dose-dependent response that requires high mechanical tension and substantial metabolic stress, neither of which characterizes low-load WRT (McCarthy et al., 2002). In addition, an 8-week intervention may be insufficient to elicit detectable architectural changes using ultrasound in athletes with advanced training backgrounds (Häkkinen et al., 2000). Accordingly, WRT appears to enhance the quality of muscle contraction, reflected in impulse and rate of force generation, rather than increasing muscle quantity, reflected in cross-sectional area or maximal structural force.

This study has several limitations. The sample consisted exclusively of male collegiate soccer players, which limits generalizability to female athletes or elite professionals. In addition, neuromuscular assessments were restricted to the vastus lateralis, which may not fully represent the coordinated multi-muscle demands of soccer-specific sprinting, jumping, braking, and change-of-direction actions (Elliott-Sale et al., 2021). Moreover, the seated knee extensor isometric RFD assessment reflects local rapid torque production and may not fully represent whole-body rapid force production during soccer-specific actions. Although participants were stratified before randomization and no significant baseline differences were observed, baseline-adjusted models were not included in the primary analysis; therefore, the borderline RFD finding should be interpreted cautiously. In addition, the ultrasound assessment was limited to vastus lateralis muscle thickness and pennation angle; muscle cross-sectional area and fascicle length were not measured. Therefore, the present findings cannot determine whether WRT induced broader changes in muscle size or fascicle architecture. Given the multi-joint nature of soccer movements, a broader electromyographic analysis including the hamstrings and gluteal muscles would provide a more comprehensive understanding of neuromuscular adaptations. Future research should examine the longer-term effects of WRT and systematically manipulate loading parameters such as load magnitude and proximal versus distal placement to identify optimal dosing strategies. The use of advanced neuromuscular assessment techniques, including high-density surface EMG and transcranial magnetic stimulation, may further clarify central and peripheral mechanisms underlying performance improvements. Moreover, direct comparisons between WRT and traditional power-oriented training modalities, such as plyometrics or weightlifting derivatives, would help contextualize its role within comprehensive athletic development programs (Hammami et al., 2019).

### 4.1 Practical applications

From an applied perspective, WRT provides strength and conditioning professionals with a validated and time-efficient micro-dosing strategy. Integrating a 2% body mass load into routine technical and tactical field sessions allows practitioners to apply a targeted neuromuscular overload without requiring additional gym training. Because this intervention improved explosive performance without significantly increasing sRPE-derived perceived internal training load, it may be suitable for integration into congested competitive periods. However, because markers of external mechanical load, muscle damage, central fatigue, and recovery status were not assessed, conclusions regarding fatigue-related outcomes should be interpreted cautiously. Coaches can implement low-load WRT to improve performance while preserving sport-specific movement quality and maintaining player readiness for competition.

## 5 Conclusion

An 8-week in-season wearable resistance training program using a 2% body mass load effectively enhanced multidirectional explosive performance in trained collegiate soccer players. Improvements in sprinting, jumping, and COD speed, together with increased rate of force development and unchanged maximal strength, vastus lateralis muscle thickness, and pennation angle, indicate that low-load WRT may primarily promotes rapid neuromuscular adaptations rather than detectable changes in the ultrasound-derived morphological measures assessed in this study. early-phase neural adaptations. Therefore, 2% body mass WRT may represent an effective in-season training strategy to improve explosive performance while maintaining a similar perceived internal training load.

## Data Availability

The raw data supporting the conclusions of this article will be made available by the authors, without undue reservation.
