# Supplementary material for: The effects of 8-week wearable resistance training on neuromuscular adaptations in soccer players
Source: Front Physiol. 2026 Jul 28;17:1828654. doi: 10.3389/fphys.2026.1828654 (PMC13457027; doi:10.3389/fphys.2026.1828654)
Supplement: Supplementary file 1 [file SupplementaryFile1.docx]

Supplement 1. Overview of the 8-week wearable resistance training intervention protocol

| **Training component** | **Weeks 1–2** | **Weeks 3–4** | **Weeks 5–6** | **Weeks 7–8** | **Intensity and rest control** |
| --- | --- | --- | --- | --- | --- |
| Weekly frequency | 3 sessions/week | 3 sessions/week | 3 sessions/week | 3 sessions/week | Monday, Wednesday, and Friday |
| Weekly WRT exposure | ~90 min/week | ~90 min/week | ~90 min/week | ~90 min/week | 3 × ~30 min sessions |
| Session timing | After standardized warm-up and before main team training | Same as Weeks 1–2 | Same as Weeks 1–2 | Same as Weeks 1–2 | 15-min standardized warm-up before WRT block |
| Wearable load | 2% body mass | 2% body mass | 2% body mass | 2% body mass | 1% body mass on proximal thighs and 1% on distal calves |
| Monday focus | Acceleration and deceleration | Acceleration and deceleration | Acceleration, deceleration, and re-acceleration | Match-specific acceleration and braking | Maximal or near-maximal effort; technical quality emphasized |
| Monday acceleration drills | 2 sets × 4 reps of 10–15 m starts | 3 sets × 4 reps of 10–20 m starts | 3 sets × 5 reps of 10–20 m starts | 4 sets × 4 reps of 10–20 m starts from varied starting positions | 30–45 s between reps; 2 min between sets |
| Monday deceleration drills | 2 sets × 4 reps of 10 m sprint-to-stop | 3 sets × 4 reps of 10–15 m sprint-to-stop | 3 sets × 4 reps of 15 m sprint-to-stop and stop-go actions | 3 sets × 5 reps of 15–20 m sprint-to-stop and re-acceleration actions | 45–60 s between reps; 2 min between sets |
| Wednesday focus | Change of direction | Change of direction and cutting | Multidirectional change of direction | Reactive and match-specific change of direction | Maximal or near-maximal effort; correct foot placement and trunk control emphasized |
| Wednesday COD drills | 2 sets × 4 reps of 5-0-5 and 5–10–5 shuttle drills | 3 sets × 4 reps of 5-0-5, zig-zag, and 90° cutting drills | 3 sets × 5 reps of zig-zag, 90°/135° cutting, and 180° turning drills | 4 sets × 4 reps of reactive shuttle, cutting, and 180° turning drills | 45–60 s between reps; 2–3 min between sets |
| Friday focus | Small-sided games and multidirectional actions | Small-sided games and repeated acceleration/deceleration | Higher-density small-sided games | Match-specific small-sided games | Coaches maintained high intensity using standardized encouragement and rapid ball replacement |
| Friday small-sided games | 4 vs 4, 4 × 3 min bouts | 4 vs 4 or 5 vs 5, 4 × 3 min bouts | 5 vs 5, 4 × 4 min bouts | 5 vs 5, 4 × 4 min bouts | 2 min passive recovery between bouts |
| Pitch size during SSG | ~25 × 20 m | ~25 × 20 m to 30 × 25 m | ~30 × 25 m | ~30 × 25 m | Pitch size kept identical between groups within each session |
| Progression strategy | Familiarization with loaded movement and technical control | Increased total repetitions and sprint distance | Increased movement density and COD complexity | Increased reactive and match-specific movement demands | Relative load remained constant at 2% body mass |
| Intensity monitoring | sRPE collected 30 min post-session | Same as Weeks 1–2 | Same as Weeks 1–2 | Same as Weeks 1–2 | sRPE × session duration used to calculate internal training load |
| Adherence monitoring | Attendance recorded each session | Attendance recorded each session | Attendance recorded each session | Attendance recorded each session | Session completion and absences recorded by coaching staff |
| Control group | Identical training with unloaded garments | Identical training with unloaded garments | Identical training with unloaded garments | Identical training with unloaded garments | Same duration, drills, rest intervals, pitch dimensions, and coaching supervision |
